# Exploring Suicidal Ideation Using an Innovative Mobile App-Strength Within Me: The Usability and Acceptability of Setting up a Trial Involving Mobile Technology and Mental Health Service Users

**DOI:** 10.2196/18407

**Published:** 2020-09-28

**Authors:** Ashley Jane Bruen, Abbie Wall, Alina Haines-Delmont, Elizabeth Perkins

**Affiliations:** 1 Department of Primary Care and Mental Health University of Liverpool Liverpool United Kingdom; 2 Department of Nursing, Faculty of Health, Psychology and Social Care Manchester Metropolitan University Manchester United Kingdom

**Keywords:** mobile applications, smartphone, mobile phone, mHealth, mental health, suicide, social media

## Abstract

**Background:**

Suicide is a growing global public health problem that has resulted in an increase in the demand for psychological services to address mental health issues. It is expected that 1 in 6 people on a waiting list for mental health services will attempt suicide. Although suicidal ideation has been shown to be linked to a higher risk of death by suicide, not everybody openly discloses their suicidal thoughts or plans to friends and family or seeks professional help before suicide. Therefore, new methods are needed to track suicide risk in real time together with a better understanding of the ways in which people communicate or express their suicidality. Considering the dynamic nature and challenges in understanding suicide ideation and suicide risk, mobile apps could be better suited to prevent suicide as they have the ability to collect real-time data.

**Objective:**

This study aims to report the practicalities and acceptability of setting up and trialing digital technologies within an inpatient mental health setting in the United Kingdom and highlight their implications for future studies.

**Methods:**

Service users were recruited from 6 inpatient wards in the north west of England. Service users who were eligible to participate and provided consent were given an iPhone and Fitbit for 7 days and were asked to interact with a novel phone app, Strength Within Me (SWiM). Interaction with the app involved journaling (recording daily activities, how this made them feel, and rating their mood) and the option to create safety plans for emotions causing difficulties (identifying strategies that helped with these emotions). Participants also had the option to allow the study to access their personal Facebook account to monitor their social media use and activity. In addition, clinical data (ie, assessments conducted by trained researchers targeting suicidality, depression, and sleep) were also collected.

**Results:**

Overall, 43.0% (80/186 response rate) of eligible participants were recruited for the study. Of the total sample, 67 participants engaged in journaling, with the average number of entries per user being 8.2 (SD 8.7). Overall, only 24 participants created safety plans and the most common *difficult emotion* to be selected was feeling sad (n=21). This study reports on the engagement with the SWiM app, the technical difficulties the research team faced, the importance of building key relationships, and the implications of using Facebook as a source to detect suicidality.

**Conclusions:**

To develop interventions that can be delivered in a timely manner, prediction of suicidality must be given priority. This paper has raised important issues and highlighted lessons learned from implementing a novel mobile app to detect the risk of suicidality for service users in an inpatient setting.

## Introduction

### Background

Suicidality has been defined as any suicide-related behavior, including completing or attempting suicide (intent), suicidal ideation (thoughts), or communications [1]. Suicide is a growing public health concern, with 6507 suicides registered in the United Kingdom in 2018 [2]. However, it has been suggested that suicide rates are often underestimated [3], raising questions about the reliability and accuracy of statistics related to suicide [4]. A recent report examined the current trends in suicide rates in the United Kingdom and reported that suicide is more prevalent among men aged 45-49 years, with men 3 times more likely to take their own lives than women [5]. The rate of suicide among young people between the ages of 15 and 24 years has risen since 2013, with the suicide rate for young women now at its highest on record (9.5 per 100,000). It is argued that many suicide deaths can be prevented [6].

However, although the global prevalence of mental health disorders in the general population is high, the use of mental health services is comparatively low [7]. Research has identified barriers, including stigma, lack of time, and pragmatic issues of accessing services which often prevent people who have mental health problems and who are at an increased risk of suicide from seeking help and support from services [8,9]. However, the demand for National Health Service (NHS) psychological services has been increasing [10]. Inadequate resources, both staffing and financial, have resulted in 1 in 10 patients experiencing a wait of over 1 year before receiving any form of treatment [11,12]. One in 6 of those on a waiting list for mental health services is expected to attempt suicide [11]. In addition, it has been highlighted that these services often fail to provide effective, timely interventions at the point of crisis, and their traditional methods (clinical) to identify suicide risk have been criticized for lacking accuracy [8,13,14]. A systematic review revealed that risk assessments can lack validity and are not always able to predict suicidality in high-risk populations [15]. Such assessments are time consuming and may not add value to either the patient or the clinician [16].

Currently, approximately 95% of households in the United Kingdom own mobile phones, a figure that has remained constant since 2015 [17]. It is not surprising that the use of smartphone apps for research and clinical care in mental health has become increasingly popular [18]. The use of smartphones to support mental health has the potential to reach and engage with those groups of people who might find it hard to attend services [19] and has the potential to provide immediate support. Effective mental health apps may, therefore, have the ability to improve patient outcomes [20-22]. Logically, mental health apps are seen as a cost-effective and scalable solution to the gap in mental health services [23]. Unfortunately, the pace of research has not kept up with the advances in mobile technology [24], with the majority of mental health apps available for download not supported by evidence-based research and perhaps not even following treatment guidelines [25].

A review of mobile health apps for the most prevalent mental health conditions [26] identified that, of the 1500 depression-related apps in the market, only a small proportion (2%) had been tested. There is a clear need for more research to be conducted on the use, reliability, and efficacy of apps in the field of mental health [27]. Researchers interested in conducting studies focusing on suicide risk or prevention are also faced with additional methodological constraints such as ethical or safety issues [1,28]. It has been highlighted that individual-based naturalistic studies are the best for identifying prognostic factors of suicide risk [29]. Although suicidal ideation has been shown to be linked to a higher risk of death by suicide [30], not everybody openly discloses or communicates their suicidal thoughts and/or plans to friends and family or seeks professional help before suicide [9,31]. Therefore, new methods are needed to track suicide risk in real time [32], together with a better understanding of the ways in which people communicate or express their suicidality [31].

Considering the dynamic nature of and challenges in understanding suicide ideation and suicide risk, mobile apps could be better suited to prevent suicide as they have the ability to collect real-time data, thus offering support at the time of crisis [22]. Research indicates that changes in mental health symptoms can be identified by analyzing certain patterns of smartphone use [33]. Machine learning (ML) is potentially one way to expand our understanding of people’s thoughts, feelings, and behavior and to improve the monitoring of suicide risk in real time. ML uses computational methods to analyze past information to make accurate predictions [34]. A pilot study using data from 144 patients with mood disorders suggests that ML algorithms using previous clinical data were successful in distinguishing between people who attempted suicide and those who did not, with a prediction accuracy between 65% and 72% [13]. Similarly, a study that used ML from unstructured clinical notes was able to estimate the risk of suicide with an accuracy consistently ≥65% [16].

Social media is another means of collecting real-time information. It has been reported that social media has the potential to prevent suicide or identify suicide risk based on an individual’s self-expression by analyzing their status updates through the use of ML [34], thus providing clinicians with timely information for early intervention. Another study demonstrated the utility of social media blog post analysis in classifying individuals with a high suicide risk in China [35]. Sleep problems have also been identified as a risk factor for individuals with suicidal tendencies [36,37]. Specifically, a shorter sleep duration on weekdays and a longer sleep duration on weekends predicts a high risk of suicidality [38]. Unfortunately, most studies rely on self-reported measures of sleep [39], which do not provide reliable, real-time information. The use of connected sensors (wearables) offers the opportunity to collect data during sleep, producing real-time information.

This study adds to the literature by introducing external, user-generated input and smartphone data and combining them with clinical data.For the focus of this paper, we report the practicalities of implementing a study designed to test the feasibility of using ML algorithms to identify suicidal ideation and eventually predict suicide risk in acute inpatient mental health settings in the United Kingdom using a novel smartphone app–Strength Within Me (SWiM). The results of the multiple ML algorithms that were tested were reported in a previous paper [40]. The SWiM feasibility study aimed to (1) determine the degree to which participants accept and engage with the SWiM app; (2) collect sufficient data to inform the development of risk algorithms; and (3) gather participants’ feedback regarding the SWiM app, risk assessments, and participation in the study.

### The SWiM App

SWiM is a novel phone app that has been developed to provide clinicians with additional information that would otherwise not be available to them. It should be emphasized that the SWiM app is not a therapeutic intervention and was developed to improve the understanding and identification of suicide risk and, in the long term, potentially contribute to a reduction in suicide rates.

The app allows service users to journal at any time of the day using free text only; this may include a short note about what the user did that day or longer entries about their thoughts, feelings, and experiences. Participants were encouraged to record at least one journal entry per day, but the number of journal entries they could input was unlimited. When a service user completes a journal entry, there is an automatic prompt to rate their current mood using emoticon faces.

When participants sign up at baseline and when they stop using the app or are discharged from the study, they are asked to answer 4 questions about their mood and sleep during the past 7 days; these include feeling depressed, feeling hopeless, unable to fall asleep, and waking up frequently at night.

**Figure 1:**
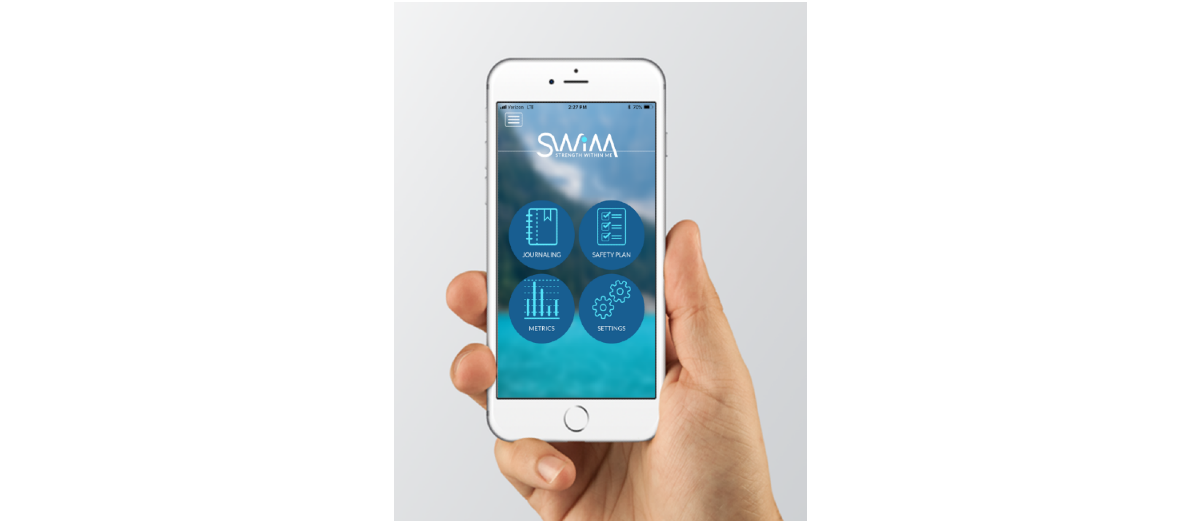
Screenshot to illustrate the front screen of the Strength Within Me app.

In addition, the SWiM app enables service users to create a safety plan using a 3-step process. Step 1 involves self-soothing activities that they can perform on their own; step 2 involves activities they can perform with a friend or caregiver if step 1 does not help; and step 3 involves identifying a professional they can contact, such as their doctor or clinician, if the first 2 steps do not help. Participants can choose from 4 emotions with which they may struggle (sad, angry, lonely, and worried) to create a safety plan. They also have the ‘*other’* option so that they can develop a safety plan for additional emotions. Safety plans can be adjusted to reflect what is working for each participant and what is not (Figure 1).

### Development of the SWiM Algorithm

For building the model, both active and passive data were gathered. Active data (where the user engaged with the SWiM app; the data included journaling, safety plans, and rating mood) complemented the passive data collected (where the user did not actively engage with the app; the data included social media [Facebook posts], sleep monitoring [sleep quality], and daily activity [number of steps], as recorded by the participant wearing a Fitbit). Unfortunately, social media data were excluded from the model because of the low number of participants who consented to the use of Facebook data. The data were analyzed to train the ML algorithms to produce a risk score that deduced the likelihood of suicide, which was analyzed using natural language processing (NLP). This involved extracting language patterns to make inferences about people’s thoughts and feelings [41,42]. A study demonstrated that NLP analysis using language from social media posts could identify people at risk of suicide [43]. In addition, clinical data using assessments conducted by trained researchers looking at suicidality, depression, and sleep enabled comparisons to be made generating a risk score were collected.

### Aim of the Paper

This study aims to report the practicalities and acceptability of setting up and trialing digital technologies within an inpatient mental health setting in the United Kingdom and to highlight the implications of these for future studies.

## Methods

### Recruitment Procedure

As the SWiM app was novel and untested, it was important to first introduce and test it in a controlled environment for the safety of participants–in this case, vulnerable service users presenting with acute mental health difficulties. This enabled the research team to monitor for any possible side effects. It also enabled researchers and data scientists to monitor the data, keep track of equipment, and determine whether service users would actually engage with the app. Service users were recruited from 6 NHS acute adult mental health wards in the north west of England in the United Kingdom.

Service users were eligible to participate in the study provided they had the capacity to give informed consent and possessed a good command over written and spoken English. Exclusion criteria included service users who were unwell at the time of recruitment (experiencing psychosis or significant agitation), those unable to use an iPhone, those undergoing detoxification, anyone with visual or other physical impairments that would impede their use of a mobile phone or app, and service users who had been readmitted within the study time frame.

To facilitate recruitment, researchers discussed any new admissions (admitted within the last 7-10 days) with nursing staff in each participating ward and went through the inclusion and exclusion criteria. Those people who were considered by the clinical care team to be eligible to participate in the research were referred to the research team. The researchers went through the participant information sheet with each participant and recorded their written informed consent. Eligibility was continuously assessed by researchers throughout the recruitment procedure. Participants were also asked whether they would allow the study to access their Facebook data via a secondary opt in app, SMiLE. This was to allow patterns in their social media content and activity to contribute to the development of the risk algorithm(s). Participants could opt in or out of this aspect of the study without affecting their overall participation.

It had been established in the pilot work that this particular service user population did not routinely have access to an iPhone or a Fitbit; therefore, participants were loaned an iPhone and a Fitbit device. They were also asked to sign a contract form agreeing not to purposefully damage the equipment and to return it at the end of the study period. These documents were photocopied and the copies included in the patient’s health record.

Between January and November 2018, there were 810 admissions across 6 wards, yielding 186 eligible service users. Furthermore, 105 service users declined to participate, resulting in 81 who consented to participate in the study. Unfortunately, 1 service user who had consented to participate was excluded because of the recruitment website not working. This resulted in 43.0% (80/186 response rate) participants taking part in the study, with 66 included in the analysis based on the completion of at least two follow-up clinical assessments. Reasons for noncompletion of assessment tools at all time points were as follows: declined, not available at the time of follow-up, or discharged from the ward.

### Procedure

After obtaining informed consent, participants were given a study iPhone and Fitbit for use during the following 7 days. The setup for participation in the study involved a series of technical and time-consuming procedures. One of the researchers created a new private SWiM and Fitbit account for the service user. In addition, the researcher recorded demographic data for every participant, including age, gender, weight, and height. The SWiM and Fitbit apps were configured for the service user on the iPhone and participants were shown how to access and use them.

When this was being done, the other researcher asked participants to complete a number of clinical assessments. The baseline assessments comprised 3 questionnaires that recorded the presence and severity of depression (Patient Health Questionnaire-9) [44], suicidal thoughts and behavior (Columbia Suicide Severity Risk Scale [C-SSRS]) [45], and sleep difficulties (Insomnia Severity Scale) [46]. Questionnaires were completed on 2 additional occasions: at follow-up 1 (approximately 3 days later) and at follow-up 2 (approximately 3 days after follow-up 1 or at the end of the study). The expectation was that service users would participate in the study for a maximum period of 7 days. If they were discharged within the 7-day study period from the ward, the staff collected the iPhone and Fitbit, and the researchers aimed to collect their final questionnaire data. At each time point, a copy of the C-SSRS was given to the staff if they had scored above 0 to ensure that each patient’s suicide risk was documented. An additional setup process was required if service users consented to the monitoring of their Facebook accounts. Service users had to input their Facebook credentials into a secure website (SMiLE).

In recognition of their involvement, shopping vouchers valued at £25 (US $32) were given to participants following completion of the assessments at baseline and at the end of the study. Participants were given the researchers’ study telephone number so that they could contact if they had any questions, problems, or required technical support. Once a participant had completed their time in the study, the researchers synced the Fitbit data, terminated their SWiM app account, and then reset both iPhone and Fitbit, which resulted in deleting all participant data from the study devices.

Some qualitative data were also obtained via a brief informal exit interview. Questions related to what participants thought of the study and the SWiM app, including likes or dislikes and improvements for future research, were asked. Notes were taken during this discussion and were analyzed thematically to illustrate their views.

### Participant Characteristics

Of the 80 participants who were recruited to the study, 35 (44%) were male and 45 (56%) were female. The mean age was 36.8 years (SD 11.6) and the age range was from 18 to 61 years.

A total of 79 participants completed the C-SSRS at baseline and all reported that they had experienced suicidal thoughts at some point in their life. There were 68 participants who reported having made at least one previous suicide attempt.

## Results

### Acceptability and Engagement of the SWiM App

#### Journaling

Overall, 653 journal entries were recorded. Of the total sample (n=80), there were 67 (84%) participants who engaged in journaling, with an average number of entries per user being 8.2 (SD 8.7). The total number of journal entries per user ranged from 1 to 43. Interestingly, the average number of journal entries for female participants was 7.2 (SD 7.1; range 1-32), whereas male participants had an average of 9.4 (SD 10.4; range 1-43) journal entries each. Participants aged between 40 and 49 years completed more journal entries on average than other age groups (Table 1).

Participants used the journaling facility on the SWiM app to report a wide range of situational and emotional factors that affected their everyday lives in the inpatient setting. The journal entries varied from a couple of words to an extensive narrative:

Feel low

Participant number 901122

Today has been mostly spent in bed. I had a injection forced upon me today which I refused several times but they left me with no choice. I have now been prescribed meal replacement drinks which I’m refusing to have. I’ve had a lot of teary moments today as I have asked for some of my personal belongings yet I was refused.

Participant number 901127

**Table 1:** Number of journal entries for each age category.

Age category (years)	Participants per age category, n	Average number of journal entries	Range of journal entries
18-29	24	10	1-32
30-39	22	7	1-24
40-49	22	12	1-43
50-59	10	11	3-21
60-64	1	1	1

#### Safety Plan

Overall, only 30% (24/80) participants created safety plans. A total of 61 safety plans were completed, with an average of 2.5 plans per person (SD 1.4; range 1-5). The most common emotion that participants completed a safety plan for was feeling sad (n=21). Within the safety plans, 113 helpful activities were recorded by participants, for example, talking to someone, participating in exercises, and listening to music.

#### Participant Feedback About the SWiM App

One of the main themes that emerged was how helpful the SWiM app had been. The ability to write-out thoughts suited those people who might otherwise have had to *struggle to voice these verbally* (Participant number 901127). One participant reported a preference for writing their thoughts into the journaling feature of the app as opposed to talking to staff face-to-face about their feelings. Many service users reported that they liked having the ability to openly express their thoughts in the SWiM app without having the staff always assume that they would act on their thoughts. From their reported experience, expressing their suicidal thoughts to a member of staff often resulted in the removal of privileges such as time off from the ward. The potential implications of this will be discussed later in this paper. In addition, several participants identified the ease of using an app on a phone; you “don’t always have pen and paper, but you always have your phone” (Participant number 902113). This enabled participants to engage with the app at any time of the day.

The app required participants to complete a journal entry before being given the option to rate how they were feeling. Many participants found the journal entry component of the app difficult to write, reporting that it required a level of motivation that they did not always possess. They reported that they would have preferred to have the option of rating their mood separately. It was not possible to discern, however, whether mood fluctuations acted as a trigger for accessing the app and recording those thoughts and feelings. Although the app did allow participants to review previous journal entries, which were perceived by some as being useful to monitor any progress they had made, some participants reported that this actually opened up negative thoughts again. Moreover, 1 participant commented that they were bored using the app after a few days as they had begun to feel well again and did not feel the need to use it anymore. Participants also commented on improvements they would like to see in the app, such as changing the color scheme, including uplifting quotes, having self-help links, and an SOS (an abbreviation for distress) button for helpful contact numbers.

#### Participant Feedback in Relation to Fitbit

Fitbit’s primary purpose in this study was to support the data obtained from the SWiM app by providing information regarding the participants’ sleep and daily activity. The majority of participants (n=69) provided positive responses about using Fitbit, which included increased self-awareness of levels of physical activity, goal setting, and peer motivation. Participants also reported changing their behavior, in particular, increasing their activity levels and adopting activity goals as a result of wearing Fitbit. They also reported interacting with other Fitbit wearers participating in the study to increase their activity. The use of Fitbit, therefore, had some unforeseen positive benefits. This is in line with previous findings indicating that mental health service users found Fitbit a useful and accessible form of technology [47-49].

### Practicalities of Using the SWiM App

#### Technological Issues

When service users were recruited to the study, researchers had to input their SWiM and Fitbit credentials into a secure website. This website provided participants with their own private SWiM and Fitbit accounts. There were several occasions in which researchers were unable to access this site because of updates and modifications being done, which meant that some eligible service users could not be recruited at that time, delaying recruitment. However, only 1 service user (who had consented) could not be recruited because of this issue as they were being discharged when the recruitment site was operational again. In addition, there were times when the app was not functioning correctly (ie, journals not getting saved and mood ratings not working), resulting in loss of data. Overall, this issue affected 12 participants.

The staff at the participating sites expressed concerns about giving service users iPhones and Fitbits for the duration of the study, suggesting that the equipment would either be stolen or damaged. They were also concerned about who would be responsible for monitoring the devices. However, out of 18 iPhones and Fitbits, only 1 phone and 1 Fitbit were lost and 1 phone was accidently damaged. All other devices were returned intact.

#### Relationships and Gatekeeping

We found that the relationships researchers had formed with staff at the wards were crucial for recruitment. Moreover, it was common to find that when senior staff were committed to the study (consultants, ward managers), the ward staff seemed to be more interested and proactive.

In areas of high staff turnover, the proportion of staff who did not know about the study increased and the initial stage of recruitment to the study was more difficult. The visibility of researchers at the wards increased awareness of the study and helped retain the momentum of recruitment.

Although considerable effort was expended in ensuring that the nursing staff understood the eligibility criteria, there were instances where service users who were identified by the nursing staff as eligible could not consent to the study when approached by the researchers. This was most often because of fluctuations in their mental health.

#### Facebook Use

The original plan was to monitor the use of social media by looking at Facebook usage with the participants’ consent. However, during the course of the study, Facebook changed their approach to data sharing. This meant that not all participants could be offered the opportunity to access their Facebook data. Of the 61 participants who were asked, only 13 agreed to allow their data to be accessed. Reasons for not consenting to sharing Facebook data included: not having a Facebook account (n=27), not using Facebook at the time of recruitment (n=10), unable to remember Facebook password (n=3), or simply declining (n=8). Importantly, some participants explained that they attributed their emotional distress to the use of Facebook, and other people reported that using Facebook while experiencing a deterioration in their mental health accentuated their distress. Others reported negative thoughts about themselves when they compared their lives with those of their family and friends as depicted in their Facebook posts. These reports echo those of many other researchers who report a complex, mixed, and uncertain association between social media and mental health [50,51]. The Facebook data collected were excluded from any analysis because of the low response rate.

## Discussion

### Summary

Being able to identify individuals at imminent risk of suicide is a major challenge because of the high prevalence of varying risk factors [52]. Therefore, detecting suicide risk in real time is an important part of reducing suicides [32] and understanding how people communicate or express their suicidality [31]. One way to address this is through the development of digital apps [53]; although they may have the ability to transform mental health care [23], integrating them into mental health settings for research studies can be complex and often underestimated. As demonstrated in this study, when mobile technologies are used in the context of mental health, there are some challenges. In this paper, we have reported some aspects of the study involving the development of an innovative mobile app, SWiM, that worked well and some issues that created difficulties for the study. In the following sections, we examine the implications of these findings and considerations for future research.

### Technological Issues, Technology Damage, and Associated Costs

One of the advantages of using digital technology is that it has the potential to be an efficient and cost-effective approach to treating mental illness [20]. In general, apps are openly accessible and the use of an app by 1 individual does not prevent another from using the same service at the same time [10]. They provide constant availability and greater access to support [25]. However, there are cost issues that might hinder the adoption of health apps within services such as access to smartphones, connectivity, development and regular updates of apps, and the maintenance of the technology [23].

This study found that 4G connectivity was essential for reliable and continuous access to the SWiM app. An important element in this study was capturing people’s thoughts in real time and fluctuations in mood. This required participants to be able to journal at any time without the worry of loss of connectivity and journal entries not being saved. Future studies using mobile apps should strive for as much connectivity as possible to allow for consistent functionality [54]. Although this may have financial implications for research budgets, it is a vital consideration for future research not only to ensure continued participation in studies, particularly if the app is to be used in people’s homes in the community, but also for security reasons. This is particularly important when the connectivity cost of downloading an app is charged to the user. This was highlighted in a study that suggested that some patients may not be able to afford the cost of the required internet connection to run an app, which may act as a deterrent to its use [55]. In addition, the requirement to update apps may also pose a significant burden in both time and effort on service users.

Of the total sample, 12 participants were affected by technological issues, with 1 service user being unable to take part. This study illustrates the importance of having the research team available in participating wards to trouble shoot issues in a timely manner. It also demonstrates the significance of having positive working collaborations within the research team, including data scientists and technicians, to ensure a continuous flow of data. This is supported by previous research that emphasizes the importance of collaborative partnerships between researchers, clinicians, app developers, and service users [56].

The initial cost of the devices and the possibility of having to replace them were concerns in this study that proved to be unfounded. A concern raised by staff in the context of this study was that smartphones would be broken, lost, or stolen [57]. Clear policies about responsibilities and implementation could help avoid these issues [49]. In this study, a contract with the service user placed the responsibility for the iPhone and Fitbit on the participant. The extent to which the contract influenced how a participant took care of the devices is unclear. It is possible, in general, that people are more responsible about other people’s equipment than we give them credit for.

### Relationships and Gatekeeping

In general, research within the NHS is highly reliant on ward staff and clinician support, and the design of this study required staff to identify eligible patients for the researchers to approach and consent. When the clinical care teams are already busy and their role in research is seen as an addition to their work, it is likely that recruitment might not always happen in the same way over time [58,59]. Staff may be too busy to prioritize research and may not see participation as integral to their role [60,61]. Likewise, previous research has reported that clinicians have expressed difficulty in maintaining enthusiasm for research with other responsibilities they had [62].

Establishing good working relationships between research and clinical staff before commencing recruitment and then maintaining them is vital to building enthusiasm for study trials [61]. In line with this, the value of developing positive working relationships with frontline workers is equally important. Although having an initial set-up meeting was critical for the study to begin, having constant negotiations with the staff maintained the profile of the study with staff. It has been suggested that key engagement strategies should be employed with frontline staff in the design and rollout of a study to improve engagement [53]. Similarly, it has been reported that positive relationships are crucial for effective recruitment [63]. Future research should consider the most effective ways of engaging with the wider clinical team to enhance recruitment to studies.

### Apps Versus Face-to-Face Contact

The use of phone apps within mental health care is on the rise, with more people preferring to communicate in this manner [18,23]. However, 1 recent study surveyed college students and found that only 26% reported that they would use mental health apps, with the majority (81%) preferring to talk to a person [64]. It has also been suggested that digital apps can help reduce barriers to face-to-face help-seeking, such as stigma and discomfort about discussing one’s own mental health [65]. Feedback from the current study suggested that some participants enjoyed using the SWiM app because they were able to write their thoughts down without the feeling of being judged or misinterpreted. It also enabled some participants to express thoughts and emotions that they did not feel comfortable discussing with the staff.

There are important questions about how the data derived from digital apps compare with those elicited using face-to-face contacts. In this study, several participants reported that they experienced suicidal thoughts on which they had no intention of acting. This raises the question of whether in clinical practice there is a tendency to over-rely on personal expression of suicidal thoughts, which may lead to premature and unnecessary intervention in the lives of service users. In 2017, Facebook reported using artificial intelligence to detect the part of a Facebook post or video that matched suicide risk patterns. Anyone expressing thoughts of suicide in any type of Facebook post would trigger a response by prevention-trained human moderators [66]. Given the findings of the study reported in this paper, careful consideration needs to be given to the ways in which people think and feel in the context of suicide if digital apps are to be designed to accurately predict the risk of suicide.

### Suicidal Risk and Facebook Use

Social networking has become embedded in the everyday lives of a large proportion of the population [67]. Facebook remains the largest and most popular web-based social networking site worldwide and has been estimated to have over 2.4 billion active members [68]. With this consideration, there was an assumption that the majority of eligible participants for the study would actively use Facebook. In reality, this was not the case. A study exploring the difficulties experienced by 28 people with depression and anxiety when using the internet and social media sites such as Facebook found just over one-third of the sample reported Facebook to be the site with which they had most difficulty, similar to this study [69]. Reasons included exposure to unexpected, inappropriate content that caused them to feel upset and frustrated, exposure to content that might trigger negative memories, social comparison cues including comparing their present with the past, difficulties with direct contact, and the pressure to maintain social networking. As the number of service users actually using Facebook in our study was low, there needs to be careful consideration for further research regarding other potential markers that use real-time information that might add to the prediction of suicide risk.

### Age and Mental Health Apps

Previous research on mobile health apps [18,70] found that there seems to be a strong emphasis on younger adults’ mental health because of the high prevalence of ownership and access to smartphones in this age group. Furthermore, it has been suggested that older people in particular may have a lack of knowledge, discomfort, and difficulty in operating new technology devices [71-73].

In contrast to previous research that suggests older people may have difficulties with mental health apps, this study included people aged 18 to 61 years, and there were no reports from participants about difficulty in using iPhones or Fitbits. In a study of US veterans [70], it was found that although age can represent a barrier to owning a smartphone, once a person has access to a device, age was not correlated with interest in or usage of mental health apps. Older people were not less interested or involved with the apps than their younger counterparts.

Furthermore, the findings from this study report positive feedback about the usability of the SWiM app from participants across all ages. As age does not appear to be a factor that affects a participant’s interest or ability to use mental health apps, research in digital apps should therefore not assume that older people will not be interested in participating. Given the aging population, research, using mobile phone apps should allow for the participation of adults across a wide range of age groups and should not just focus on younger people.

### Limitations

Although many of the issues raised by participants in this study may have wider applications, the inpatient context restricts the extent to which the findings can be applied to other settings, such as community settings. During our study, there were reports from participants that the SWiM app had helped them occupy their time during their stay in the hospital. The presence of researchers at the wards also provided a means of keeping the study in the minds of the participants. In this context, it may not be surprising that the majority of participants engaged with the SWiM app. In the community, however, with the distractions of everyday life and the absence of a research team to act as a reminder, it is not clear whether engagement would be so high. These are aspects of the SWiM app that would have to be assessed if the app were to be used in community populations.

The SWiM app is currently configured to operate on an iPhone only. During the initial planning phase of the study, it was assumed that service users who agreed to participate would be able to use their own iPhone to download and access the SWiM app. However, there were concerns from clinicians based at the recruitment sites that the majority of patients would not own an iPhone. Taking this into consideration, the research team completed a small, snapshot survey at one time point to establish the number of patients who owned an iPhone and found that only 9 patients across 3 wards had access to an iPhone. As a result, a decision was made to purchase iPhones to loan to the participants. If the SWiM app is to become available for download in the future, app versions catering to a range of smartphones will be required.

During the study period, there were 810 admissions across all 6 participating wards. Only 80 participants were recruited, with the majority of service users meeting the exclusion criteria. Although the sample size was sufficient for this study, the very nature of the target population may indicate that recruitment to digital trials takes longer than in other settings. The inpatient context also restricted participation to people in the age range of 18 to 64 years. More work is required to assess the acceptability of the SWiM app with younger and older populations as well as with people living in the community.

### Conclusions

The need to intervene precisely during a critical moment of potential suicidality could reduce the loss of life. However, to develop interventions that can be delivered in a timely manner, the prediction of suicidality must be given priority [39]. This paper has highlighted and raised important issues and lessons learned from trying to understand how service users express their suicidality using a novel phone app in an inpatient setting. Although our findings are restricted to an inpatient population, the practical components of trialing a novel phone app within health services may be valuable for future research and health care organizations.

## Abbreviations

C-SSRS: Columbia Suicide Severity Risk Scale
ML: machine learning
NHS: National Health Service
NLP: natural language processing
SWiM: Strength Within Me
